# The long noncoding RNA TUG1 acts as a competing endogenous RNA to regulate the Hedgehog pathway by targeting miR-132 in hepatocellular carcinoma

**DOI:** 10.18632/oncotarget.19582

**Published:** 2017-07-26

**Authors:** Jingjing Li, Qinghui Zhang, Xiaoming Fan, Wenhui Mo, Weiqi Dai, Jiao Feng, Liwei Wu, Tong Liu, Sainan Li, Shizan Xu, Wenwen Wang, Xiya Lu, Qiang Yu, Kan Chen, Yujing Xia, Jie Lu, Yingqun Zhou, Ling Xu, Chuanyong Guo

**Affiliations:** ^1^ Department of Gastroenterology, Shanghai Tenth People’s Hospital, Tongji University School of Medicine, Shanghai 200072, China; ^2^ Department of Clinical Laboratory, Kunshan First People’s Hospital Affiliated to Jiangsu University, Kunshan 215300, China; ^3^ Department of Gastroenterology, Jinshan Hospital of Fudan University, Shanghai 201508, China; ^4^ Department of Gastroenterology, Minhang Hospital, Fudan University, Shanghai 201100, China; ^5^ Department of Gastroenterology, Shanghai Tenth Hospital School of Clinical Medicine of Nanjing Medical University, Shanghai 200072, China; ^6^ Department of Gastroenterology, Shanghai Tongren Hospital, Shanghai Jiao Tong University School of Medicine, Shanghai 200336, China

**Keywords:** Hedgehog, TUG1, miR-132, hepatocellular carcinoma

## Abstract

Emerging evidence shows that the Hedgehog pathway and the long noncoding RNA TUG1 play pivotal roles in cell proliferation, migration, and invasion in tumors. However, the mechanism underlying the effect of TUG1 and the Hedgehog pathway in hepatoma remains undefined. In the present study, we showed that the expression of TUG1 was negatively correlated with that of microRNA (miR)-132, and depletion of TUG1 inhibited the activation of the Hedgehog pathway *in vitro* and *in vivo*. We showed that TUG1 functions as a competing endogenous (ceRNA) by competing with miR-132 for binding to the sonic hedgehog protein in HCC, thereby suppressing the activation of Hedgehog signaling and its tumorigenic effect. These data indicate that targeting the TUG1-miR132-Hedgehog network could be a new strategy for the treatment of HCC.

## INTRODUCTION

Hepatocellular carcinoma (HCC) is a common fatal malignant neoplasm, ranking sixth in incidence and third in mortality among cancers worldwide [1]. Recent advances in genomics and molecular cell biology have uncovered many carcinogenic factors and molecular mechanisms underlying HCC. However, the efficacy of treatments such as surgery, radiotherapy, and chemotherapy is limited because most HCC patients are diagnosed at advanced stages [2]. Therefore, elucidating the relevant molecular and cellular mechanisms of HCC is important.

The Hedgehog (Hh) gene was first discovered in Drosophila in 1980, and the Hh pathway is the classical signaling pathway associated with embryonic development and differentiation. The Hh signaling pathway involves Hh ligands (sonic hedgehog [Shh], desert hedgehog [Dhh], and indian hedgehog [Ihh]), membrane proteins (patched [Ptch], SMO, and Gas1), transcription factors (Gli1, Gli2, and Gli3), and target genes [3]. Although the Hh pathway is inactive in the mature liver, activating mutations in the Gli transcription factor in acute and chronic liver injury were recently shown to regulate cell growth [4]. Furthermore, the occurrence of malignant tumors is often associated with a high frequency mutation of this gene that leads to the unlimited proliferation of tumor stem cells. Activation of the Hh signaling pathway and sustained activation of target genes is associated with basal cell carcinoma of the skin, gastrointestinal cancer, prostate cancer, breast cancer, acute myeloid leukemia, pancreatic cancer, and lung cancer [5-8]. However, constitutive activation of the Hh pathway is rare in HCC, underscoring the need to identify the factors or molecules involved in the inactivation of Hh signaling.

MicroRNAs (miRNAs) are small (21–25 nucleotides) non-coding RNAs that regulate transcription and translation by binding to complementary sequences in the 3′ untranslated region (3′-UTR) of target mRNAs. miRNAs such as miR-124, miR-197, miR-433, miR-101, miR-200a and miR-21 play important roles in cell growth, apoptosis, invasion, proliferation, and metastasis [9-15]. Long non-coding RNAs (lncRNAs) are an important group of non-coding RNAs involved in the regulation of miRNA activity [16]. LncRNAs are transcripts of up to 200 nucleotides, and despite the fact that they do not encode proteins, they show tissue and developmental stage specific expression, suggesting that their regulation has biological significance [17, 18]. miRNA and lncRNA mediated transcriptional and post-transcriptional regulation is important for several essential gene expression modules. In addition, the interaction between miRNAs and lncRNAs plays essential roles. LncRNAs can function as competitive endogenous RNAs (ceRNAs) that interact with miRNAs to modulate target gene expression [19].

In the present study, we showed that TUG1 functions as a ceRNA to decrease the expression of the Shh protein in the Hh pathway by competing with miR-132, resulting in the inactivation of Hedgehog signaling, and we examined the underlying mechanism.

## RESULTS

### miR-132 was downregulated in HCC cells and patients and associated with Shh expression

**Figure 1:**
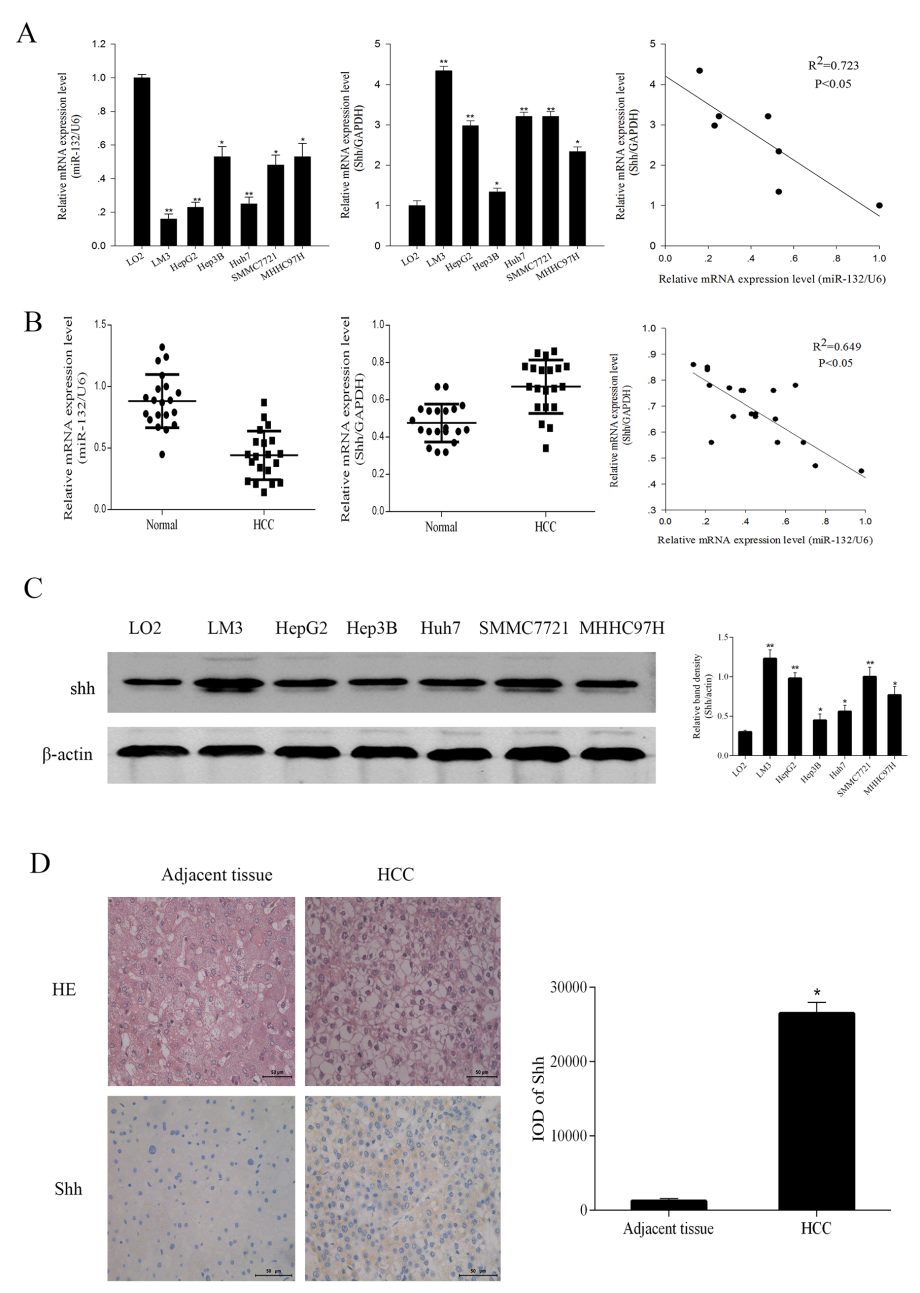
The expression of miR-132 and Shh in HCC cells and tissues **(A, B)** The relative mRNA expression of miR-132 and Shh in HCC cells and tissues was detected by qRT-PCR. Statistical analysis of the correlations between the expression of miR-132 and Shh was performed using Spearman correlation analysis in SPSS 20.0 software (R^2^ = 0.723 for cells and R^2^ = 0.649 for tissues). **(C)** The protein expression of Shh was measured by western blotting. β-actin was used as an internal control (n = 3, *P < 0.05 and **P < 0.01 for HCC cells vs. LO2). **(D)** Hematoxylin-eosin staining of HCC samples and adjacent tissues and immunoreactivity to Shh as observed by fluorescence microscopy (magnification ×400). Integrated optical density (IOD) was analyzed using Image-Pro Plus 6.0 (n = 6, **P < 0.05 for HCC vs. adjacent tissues).

miR-132 expression was examined by qRT-PCR in six HCC cell lines (LM3, HepG2, Hep3B, Huh7, SMMC7721, and MHHC97H) and one normal liver cell line (LO2) and in 20 pairs of HCC and adjacent non-cancerous tissues. The results showed that miR-132 levels were significantly lower in HCC cells than in normal hepatic cells and negatively correlated with Shh expression (R^2^ = 0.723, P < 0.05, Figure 1A). Among the HCC cell lines, LM3 and HepG2 cells showed the lowest miR-132 levels. Consistently, miR-132 expression was significantly lower in tumor tissues than in adjacent tissues concomitant with the upregulation of Shh in HCC patients. miR-132 and Shh expression were negatively correlated (R^2^ = 0.649, P < 0.05, Figure 1B). Western blot analysis and immunohistochemical analysis of biopsy specimens from HCC patients showed that Shh protein expression followed the same pattern as that of Shh mRNA (Figure 1C and 1D).

### miR-132 overexpression inhibited cell proliferation and promoted apoptosis in HCC cells

**Figure 2:**
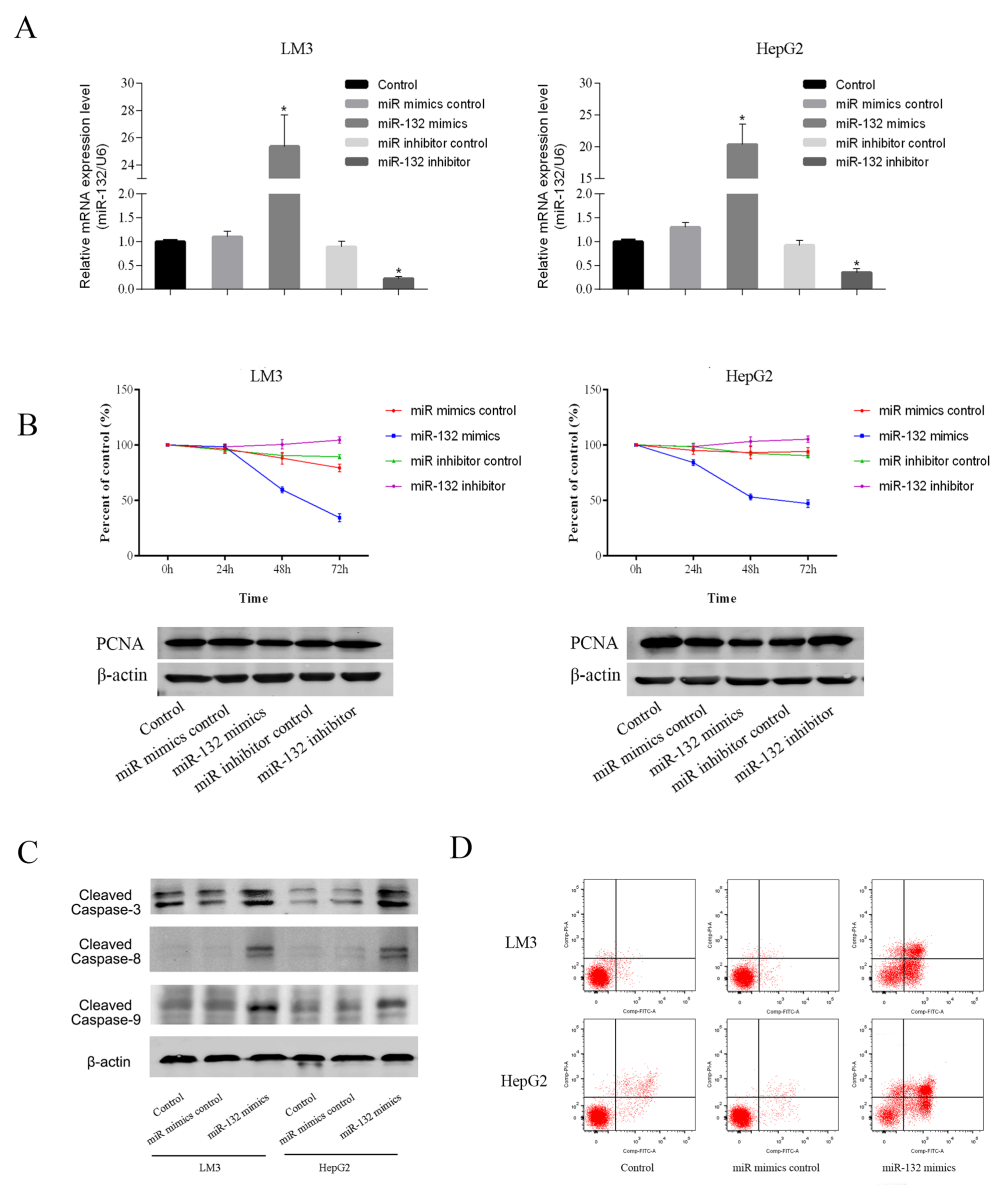
Effect of miR-132 on cell proliferation and apoptosis **(A)** The relative mRNA levels of miR-132 were determined by RT-PCR (n = 3, **p < 0.05 for miR-132 mimics or inhibitor vs. miR control). **(B)** LM3 and HepG2 cells were transfected with miR-132 mimics (50 nm) or inhibitor (100 nm). The CCK8 kit was used to monitor cell proliferation (n = 3). The protein expression of PCNA was measured by western blotting. **(C)** Apoptosis of LM3 and HepG2 cells was determined by flow cytometry and the protein expression of cleaved caspases was measured by western blotting.

To explore the functional role of miR-132, the LM3 and HepG2 HCC cell lines, which showed marked changes in miR-132 and Shh expression, were transfected with miR-132 mimics or inhibitors. After 48 h, miR-132 and Shh expression was analyzed by qRT-PCR to ensure transection efficiency (Figure 2A). The results of the CCK-8 assay showed that miR-132 overexpression significantly inhibited cell proliferation compared with that in untransfected or control mimic transfected cells. miR-132 mimics downregulated proliferating cell nuclear antigen (PCNA), an indicator of proliferation ability, at the protein level, whereas miR-132 inhibitors had the opposite effect (Figure 2B). Analysis of the effect of miR-132 mimics on apoptosis by flow cytometry showed that miR-132 overexpression increased the percentage of apoptotic cells compared with that in control cells in both LM3 and HepG2 cells. Consistently, miR-132 upregulated the expression of the apoptosis-related proteins cleaved caspases 3, 8, and 9 in both HCC cell lines (Figure 2C and 2D). These results suggested that miR-132 overexpression inhibits cell proliferation and promotes apoptosis.

### miR-132 directly targets Shh and represses Shh protein expression

**Figure 3:**
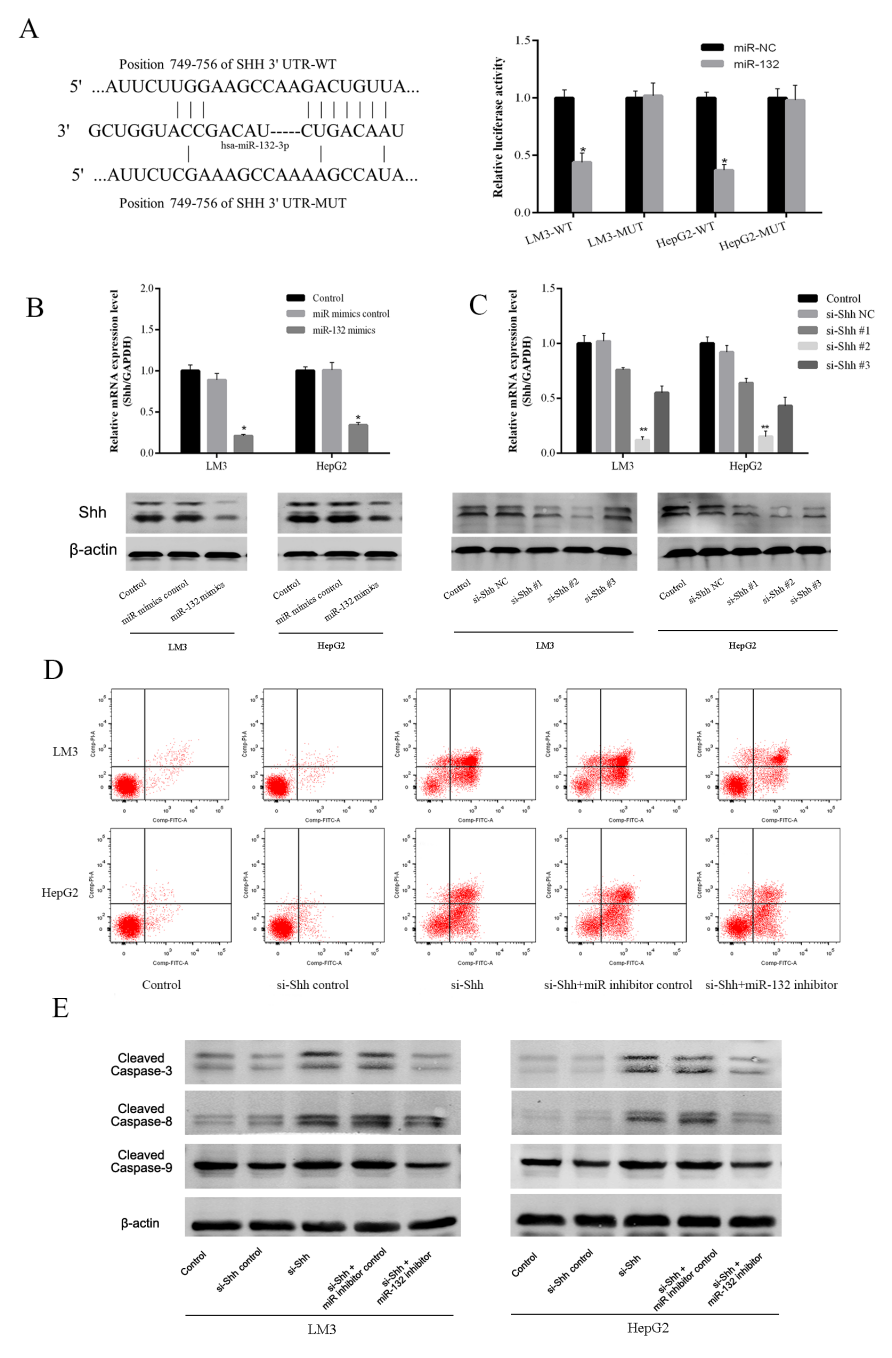
Effect of miR-132 on Shh expression **(A)** The sequences of wild-type Shh and the mutant that disrupts the interaction with miR-132 are shown. The histogram bars represent the relative dual luciferase activity in HCC cells transfected with miR-132 mimics or miR NC (n = 3, *p < 0.05 for miR-132 mimics vs. miR NC). **(B)** The relative mRNA levels of Shh were determined by RT-PCR (n = 3, *p < 0.05 for miR-132 mimics vs. miR mimics control). The protein expression of Shh was measured by western blotting. **(C)** The gene and protein expression were detected by qRT-PCR or western blotting in LM3 and HepG2 cells transfected with Shh siRNAs (n = 3, **p < 0.01 for si-Shh #2 vs. si-Shh NC). **(D)** Apoptosis of LM3 and HepG2 cells was determined by flow cytometry. **(E)** The protein expression of cleaved caspases was measured by western blotting.

To define the mechanism of action of miR-132, the Targetscan (http://www.targetscan.org/) and miRanda (http://www.microrna.org/) algorithms were used to predict miR-132 targets. Shh was predicted as a potential target of miR-132 and their interaction was confirmed using a luciferase reporter assay. After 48 h of transfection, miR-132 mimics significantly repressed the transcription of the wild-type Shh construct, whereas it had no effect on that of the mutant Shh in LM3 and HepG2 cells (Figure 3A). These results suggested that Shh is a direct target of miR-132. Assessment of Shh expression in LM3 and HepG2 cells at the mRNA and protein levels showed a similar trend as that of the luciferase reporter assay, with the downregulation of Shh by miR-132 overexpression (Figure 3B). To determine the efficacy of different Shh siRNA constructs, Shh gene and protein expression were detected by qRT-PCR and western blotting in LM3 and HepG2 cells transfected with different Shh siRNAs (Figure 3C). The results showed that Shh#3 was the most effective and this construct was used for further experiments. SiRNA-mediated Shh silencing promoted apoptosis as determined by flow cytometry, and this effect was partially reversed by miR-132 inhibitors (Figure 3D). Western blot analysis of apoptotic proteins showed similar results, as Shh silencing upregulated cleaved caspases 3, 8, and 9 and miR-132 inhibition partially reversed this effect (Figure 3E). Taken together, these results suggested that miR-132 plays a role in the regulation of the Hh pathways via the inhibition of Shh.

### TUG1 is upregulated in HCC and increases Shh protein expression by targeting miR-132

**Figure 4:**
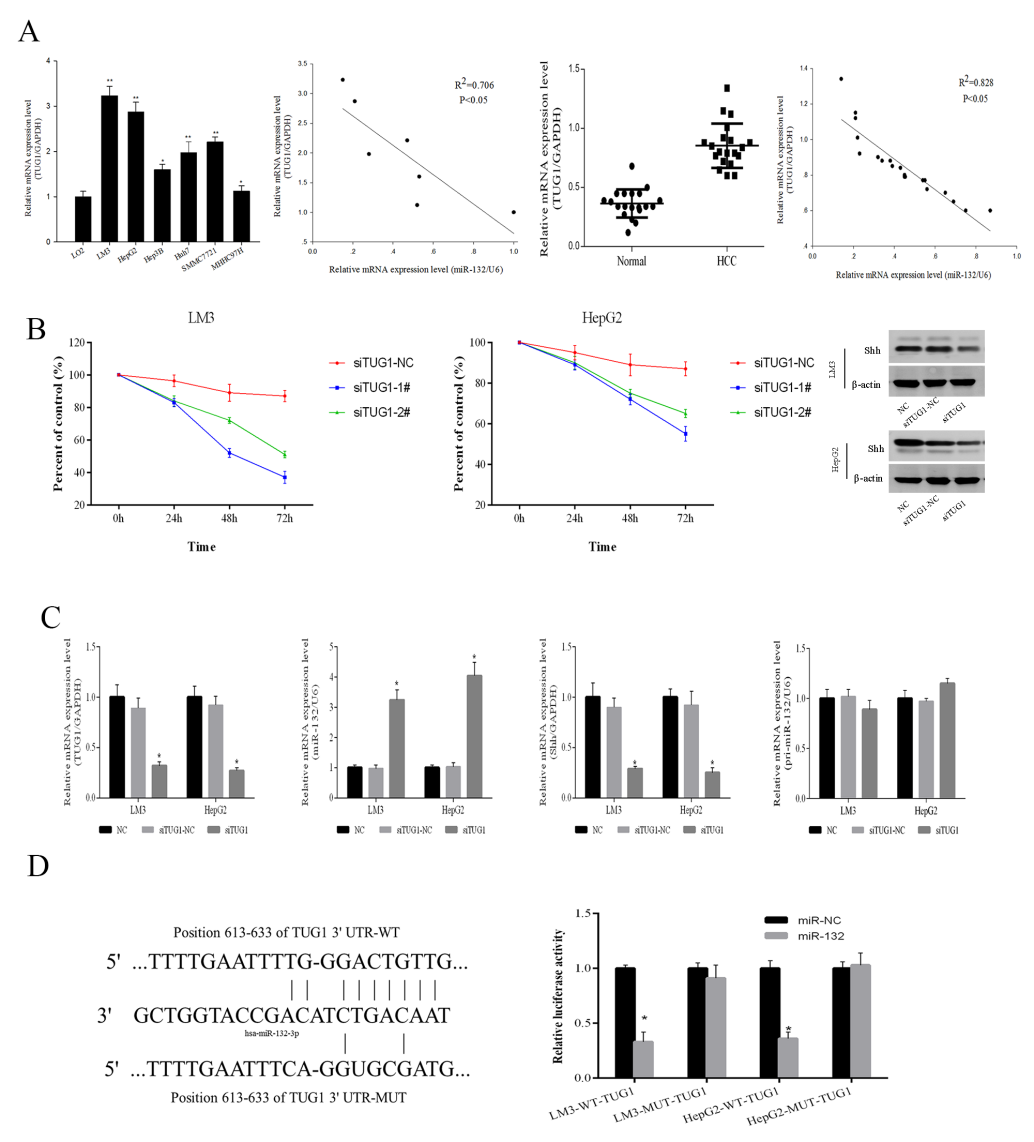
TUG1 affects Shh protein expression by targeting miR-132 **(A)** The relative mRNA expression of TUG1 and miR-132 in HCC cells and tissues was detected by qRT-PCR (n = 3, *P 0.05 and **P < 0.01 for HCC cells versus LO2). Statistical correlations between the expression of miR-132 and TUG1 were analyzed by Spearman’s test in SPSS 20.0 software (R2 = 0.706 for cells and R2 = 0.828 for tissues). **(B)** LM3 and HepG2 cells were transfected with TUG1 siRNAs (100 nm). The CCK8 kit was used to monitor cell proliferation (n = 3). The protein expression of Shh was measured by western blotting. **(C)** The expression of TUG1, miR-132, Shh, and pri-miR-132 was detected by qRT-PCR and western blotting in cells transfected with TUG1 siRNAs (n = 3, **p < 0.01 for si-TUG1 versus si-TUG1 NC). **(D)** Sequences of wild-type TUG1 and the mutant that disrupts the interaction with miR-132 are shown. The histogram bars represent the relative dual luciferase activity in HCC cells transfected with miR-132 mimics or miR NC (n = 3, *p < 0.05 for miR-132 mimics versus miR NC).

The Starbase v2.0 database (http://starbase.sysu.edu.cn/) was used to predict a potential miR-132 binding site in the lncRNA TUG1. TUG1 levels were assessed in HCC cells and tissues. As shown in Figure 4A, TUG1 expression was higher in the 6 HCC cell lines than in the normal LO2 liver cell line, and LM3 and HepG2 cells showed the most significant TUG1 upregulation. The expression of TUG1 was negatively correlated with that of miR-132 in HCC cells (R2 = 0.706, P < 0.05) as well as in 20 pairs of patient samples (R2 = 0.828, P < 0.05). siRNA-mediated TUG1 knockdown inhibited the proliferation of HCC cells within 72 h, compared with that in the negative control and downregulated Shh, as shown by western blotting (Figure 4B). The downregulation of TUG1 by siRNA transfection resulted in the upregulation of miR-132 and the downregulation of Shh, as determined by qRT-PCR (Figure 4C), indicating that TUG1 is involved in the modulation of Shh expression by miR-132 (Figure 4C). The results showing no changes in pri-miR-132 in response to siRNA-TUG1 suggested that TUG1 is a competitive endogenous RNA (ceRNA). Therefore, TUG1 may be involved in the regulation of the Hh pathway by targeting miR-132. Finally, wild-type and mutant TUG1 constructs were generated for luciferase reporter assays. The results showed that miR-132 inhibited the relative luciferase activity of the wild-type but not that of the mutant TUG1 promoter (Figure 4D). Taken together, these results demonstrated that TUG1 is upregulated in HCC cells and tissues and plays an important role in the regulation of miR-132 and Shh expression.

### TUG1 is involved in the Hedgehog signaling pathway by interacting with miR-132

**Figure 5:**
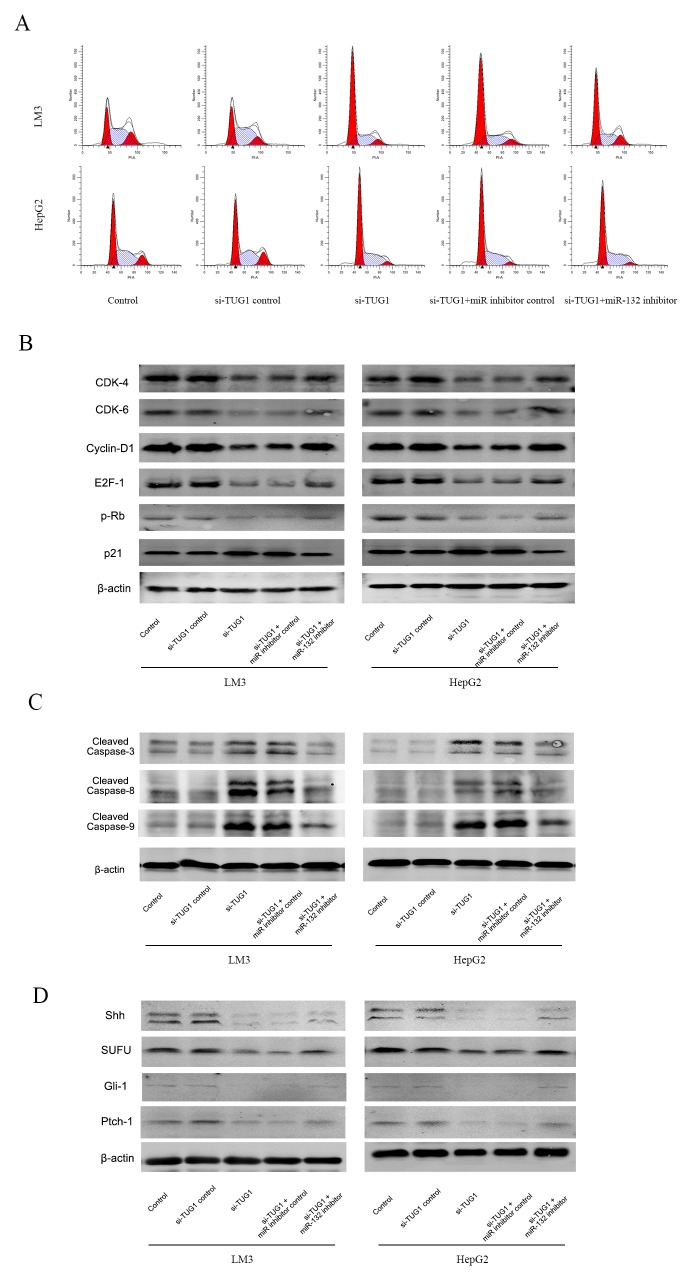
Effect of TUG1 and miR-132 on the Hedgehog signaling pathway **(A)** Cell cycle progression in LM3 and HepG2 cells was determined by flow cytometry. **(B)** The expression of proteins related to G0/G1 arrest was detected by western blotting. **(C)** and **(D)** The expression of proteins related to apoptosis and the Hedgehog signaling pathway was measured by western blotting.

To further analyze the relationship between TUG1, miR-132, and Shh and their effect on downstream pathways, cell cycle progression and the expression of downstream effectors was examined. TUG1 silencing caused cell cycle arrest in the G0-G1 phase in LM3 and HepG2 cells compared with cells transfected by siTUG1-NC (Figure 5A). Co-transfection with the miR-132 inhibitor reduced the effect to a certain extent. Western blot analysis showed that TUG1 silencing downregulated CDK4, CDK6, Cyclin D1, E2F-1, and pRb, and upregulated p21, whereas miR-132 inhibitor transfection reversed these effects, suggesting that siTUG1 repressed proliferation through miR-132 (Figure 5A and 5B). Apoptosis induced by siTUG1 showed a similar trend, as shown by the upregulation of cleaved caspases by siTUG1 and the effect of miR-132 on restoring this effect (Figure 5C and 5D). To examine the involvement of the Hh pathway, the expression of Hh pathway effectors was analyzed by western blotting. Knockdown of TUG1 downregulated the expression of Shh, suppressor of fused (SUFU), Gli-1, and Ptch-1, and treatment with the miR-132 inhibitor reversed this effect (Figure 5E).

### TUG1 inhibits tumor growth by targeting miR-132 *in vivo*

**Figure 6:**
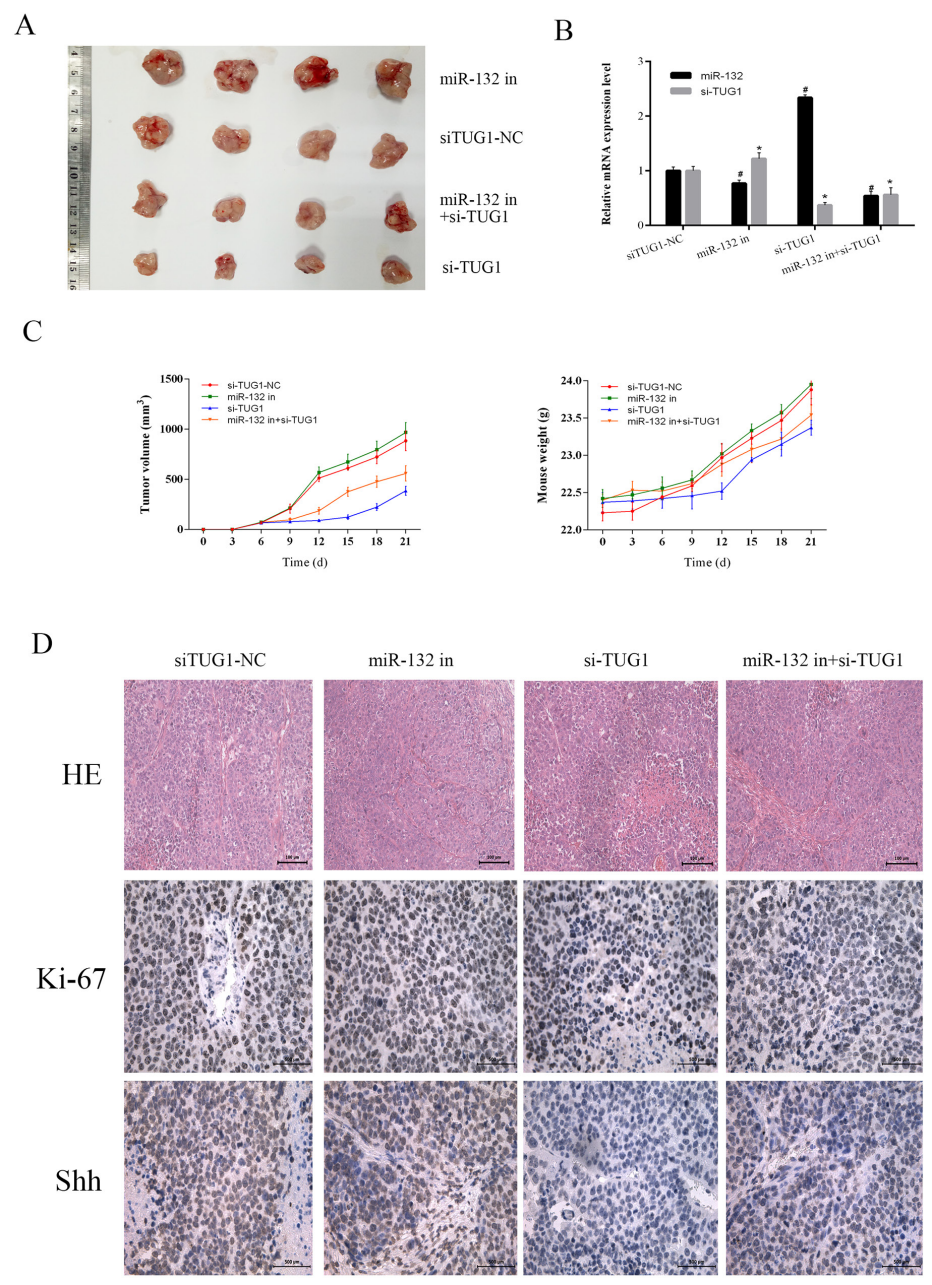
The effect of TUG1 on tumor growth *in vivo* **(A)** Gross observation of HCC-LM3 cell xenograft tumors in nude mice. **(B)** The expression of miR-132 and TUG1 were measured by qRT-PCR (n = 3, *^#^p < 0.05 for miR-132 in, si-TUG1, miR132 in+si-TUG1 vs. siTUG1-NC). **(C)** Changes in tumor volume and body weight were recorded at the time points indicated. **(D)** HE (magnification ×200) and immunohistochemical staining (magnification ×400) of tumors show the levels of Shh and Ki-67.

To verify the interaction between TUG1 and miR-132 *in vivo*, we used a xenograft mouse model generated by injection of LM3 cells transfected with siTUG1-NC, miR-132 inhibitor, siTUG1, or miR-132 inhibitor + siTUG1. The weight of mice and the diameter of tumors were monitored every 3 days. The results showed that miR-132 inhibition effectively accelerated tumor growth, whereas siTUG1 decreased tumor growth. The combination of miR-132 inhibitor and siTUG1 increased tumor growth compared with the siTUG1 group (Figure 6A and 6C). The expression levels of miR-132 and siTUG1 in tumor tissues were measured by qRT-PCR (Figure 6B). HE staining and immunohistochemical staining of Ki-67 and Shh were performed to evaluate the pathology, proliferation, and protein changes in tumor tissues. The siTUG1 group showed reduced proliferation ability and reduced Shh expression, whereas the miR-132 inhibitor reversed the effect of siTUG1 (Figure 6D). Taken together, these results indicated that TUG1 downregulated miR-132 expression to inhibit tumor growth of HCC by modulating Hh signaling *in vitro* and *in vivo*.

## DISCUSSION

For many years, scholars focused on the study of protein-encoding genes in the human genome. The rapid development of second generation sequencing technology revealed that these genes account for a small percentage of the genome, whereas non-coding RNAs account for more than 95% of the human genome [16]. miRNAs have become the focus of many studies and their involvement in liver cancer has been studied extensively [20, 21]. Although many advances have been made in improving our understanding of small RNAs, the regulatory roles and mechanisms of lncRNAs are just beginning to come to light. In the present study, we show that upregulation of miR-132 inhibits the proliferation of liver cancer cells, and we identified TUG1 as a ceRNA that interferes with the binding of miR-132 to Shh, affecting the Hh pathway and the progression of HCC.

The Hh signaling pathway plays an important role in the differentiation and development of embryos, and its excessive activation can lead to the occurrence and development of many tumors [4, 6-8]. Although the signaling mechanism underlying tumorigenesis in primary liver cancer remains unclear, an increasing number of studies show that the Hh signaling pathway plays an important role in the development and prognosis of liver cancer. Shh is the best studied ligand in the Hh pathway and it plays an important role in ectoderm development. Abnormal activation of Shh can lead to the upregulation of Myc, Ptch, and Cyclin-D, thereby promoting tumor cell division [22, 23]. Huang and Chen showed that Shh activation occurs in the early stages of HCC and increases the resistance of cancer cells to radiation therapy [24, 25]. In the present study, we predicted the relationship between miR-132 and Shh using bioinformatics software and examined their distribution and function in HCC cells and patient samples. We identified a significant correlation between low expression of miR-132 and high expression of Shh in HCC that indicated a direct or indirect relationship.

In addition, we demonstrated that miR-132 upregulation inhibits cell proliferation and promotes apoptosis, as reported previously [26, 27]. MiR-132 is a cancer-related miRNA that is upregulated in many cell carcinomas including colorectal cancer, pancreatic cancer, hemangioma, and chronic lymphocytic leukemia [27-30]. Liu et al. showed that miR-132 expression is downregulated in HCC tissues compared with adjacent noncancerous hepatic tissues and inversely correlated with HBV-related HCC [26]. We validated the direct interaction of miR-132 with Shh using a luciferase reporter gene assay and showed that miR-132 upregulation could inhibit the expression of Shh. Furthermore, co-transfection with a miR-132 inhibitor partly counteracted the effects of Shh silencing in HCC cell lines. These results indicated that miR-132 contains a sequence complementary to that of the 3′-UTR of Shh, and its binding results in Shh mRNA degradation or translation inhibition [31]. These results clarifying the relationship between miR-132 and Shh identified potential targets for the treatment of HCC.

In 2011, Salmena proposed the ceRNA hypothesis and showed that certain lncRNAs could competitively bind to miRNAs, as a supplement to the traditional theory of miRNA to RNA [32]. The role of lncRNAs as ceRNAs was reported in several studies [33-35]. Yu et al. demonstrated that MALAT1 functions as a competing endogenous RNA, modulating Rac1 expression by sequestering miR-101b in liver fibrosis, and lncRNA BC032469 was identified as a novel ceRNA that upregulates hTERT expression by sponging miR-1207-5p, promoting tumor cell proliferation in gastric cancer [36, 37]. Bioinformatics analysis showed that miR-132 shares binding sites with Shh and many lncRNAs including H19, HOTAIR, MALAT1, and CASC2. Taurine upregulated gene 1 (TUG1), a 7.1-kb lncRNA located at chromosome 22q12, was first identified in mouse retinal cells and shown to be related to miR-132. However, the exact function of TUG1 in hepatoma and its effect on the binding of miR-132 to its target gene Shh remain unclear.

**Figure 7:**
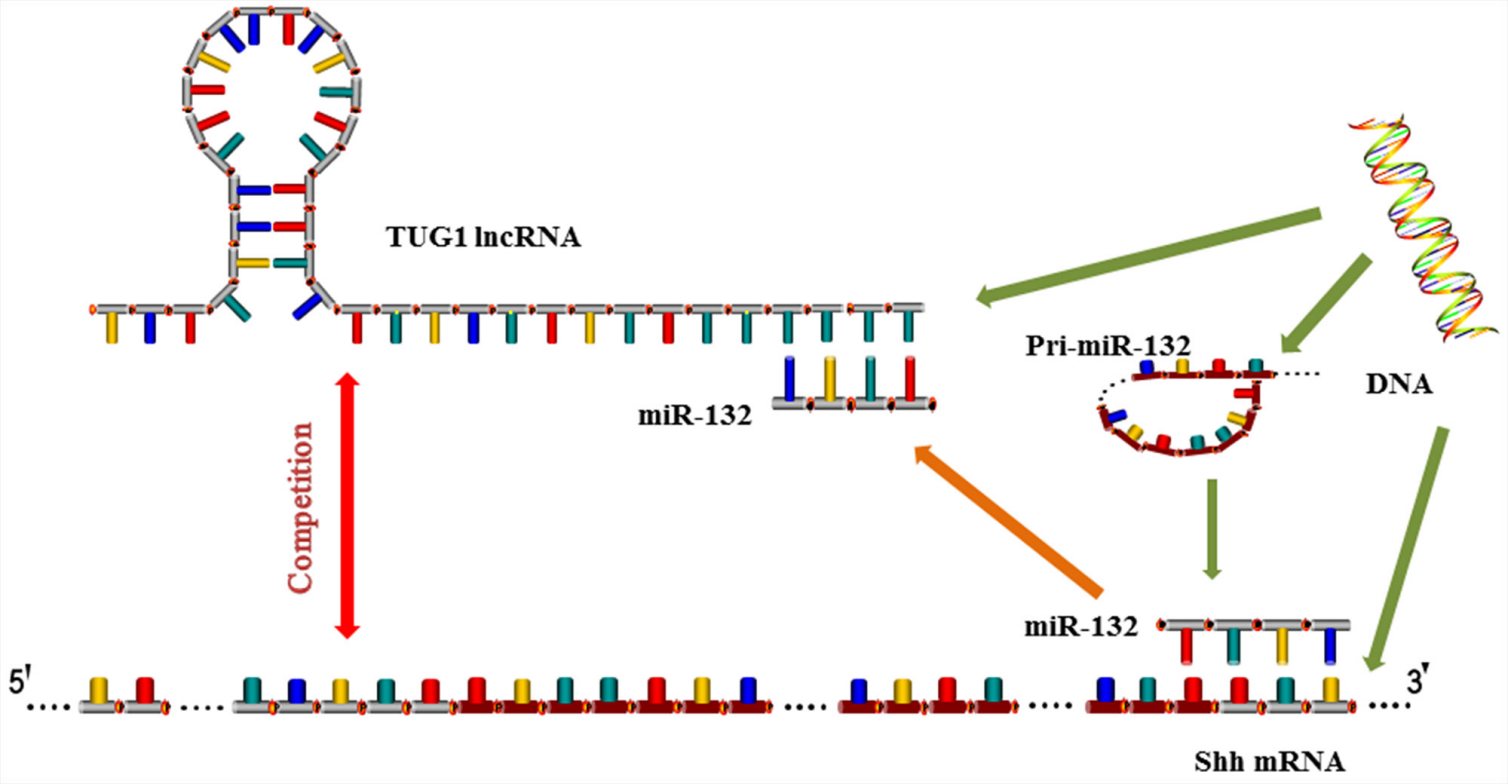
Mechanism of TUG1 and miR-132 action LncRNA TUG1 competes with miR-132 for binding to the 3′-UTR of Shh, leading to the negative inhibition of miR-132 and the activation of Hh signaling. TUG1 c acts as a “molecular sponge” and binds to miRNA response elements in miR-132, thereby inhibiting miR-132.

We therefore hypothesized that a specific lncRNA may interfere with the regulatory relationship between miR-132 and Shh. We used database predictions and previous results to identify TUG1 as a lncRNA related to miR-132 and examined the expression of TUG1 in HCC cells and tissues. The results showed that TUG1 is upregulated in HCC and its expression is significantly negatively correlated with that of miR-132, similar to Shh. Therefore, we determined whether TUG1 downregulation could effectively inhibit the proliferation of cells with increased expression of miR-132 and reduced expression of Shh. The expression of pri-miR-132 remained unchanged, indicating that TUG1 may play a regulatory role by interacting with miR-132, which was confirmed by luciferase reporter gene assays. The relationship was validated by co-transfection with siRNA against TUG1 and miR-132 inhibitors. The results showed that TUG1 downregulation caused cell cycle arrest at the G0/G1 stage and promoted cell apoptosis, which effectively counteracted the effect of inhibitors. At the same time, the downstream proteins related to the Hh signaling pathway changed accordingly. The above results indicate that TUG1 acts as a regulatory factor in liver cancer, and knockdown of TUG1 inhibits the progression of HCC through a mechanism involving miR-132 and Shh. The present results suggest a mechanism by which the lncRNA TUG1 competes with miR-132 for binding to the 3′-UTR of Shh, leading to the negative inhibition of miR-132 and the activation of Hh signaling (Figure 7) [19, 34]. An alternative explanation is that TUG1 c acts as a “molecular sponge” and binds to (miRNA response elements) MREs in miR-132, thereby inhibiting miR-132 [16, 17, 38]. In our *in vivo* experiments, changes in tumor volume and growth rate in response to TUG1 silencing further verified its regulatory effect on the growth of HCC cells.

In summary, our study indicated that TUG1 plays an important role in HCC progression by competing with miR-132 for binding to Shh, thereby acting as a ceRNA to activate the Hh signaling pathway. Targeting the TUG1-miR132-Hedgehog network may become a new strategy for the treatment of HCC.

## MATERIALS AND METHODS

### Cell culture and CCK8 assay

Human HCC cell lines (LM3, HepG2, Hep3B, Huh7, SMMC7721 and MHC97H) and one normal hepatic epithelial cell line (LO2, control) were purchased from the Chinese Academy of Sciences Committee Type Culture Collection cell bank and cultured in DMEM (Thermo Fisher, Waltham, MA, USA) containing 10% fetal bovine serum and 100 U/ml streptomycin and penicillin at 37°C in 5% CO_2_. Cells at a logarithmic growth phase were tested after being passaged every 2–3 days.

HCC cells were seeded into 96-well culture plates at a density of 5–10 × 10^3^ and 10 μl CCK8 solution (Dojido, Kumamoto, Japan) was added to each well and incubated for 3 h. At the end of the incubation period, absorbance was measured at 450 nm using a microplate reader.

The cell growth rate was calculated according to the following formula: cell growth rate = (the experimental group-the blank group) / (the control group-the blank group) ×100%.

### Tissue samples

Fresh HCC tissues and their adjacent normal samples were obtained from patients who underwent liver surgery between January 2015 and September 2016 at Shanghai Tenth People’s Hospital. Patients had not received pre- or post-operative radiotherapy or chemotherapy, and the diagnosis was confirmed by routine postoperative pathological examination following World Health Organization criteria. All patients signed an informed consent to participate in the study and the research plan was approved by the ethics committee of Shanghai Tenth People’s Hospital and in accordance with the code of ethics of the World Medical Assocition. Tissues were fixed in 4% paraformaldehyde and embedded in paraffin and sections were prepared for analysis.

### Transfection assay

miR-132 mimics, miR-132 inhibitor, Shh, and TUG1 siRNAs (RiboBio, Guangzhou, China) were stored in freeze-dried powder at −20°C and diluted with sterile RNase-free H_2_O to a concentration of 20 μm. LM3 and HepG2 cells were transfected with specific RNA oligonucleotides using Lipofectamine 3000 (Invitrogen, Carlsbad, CA, USA) according to the manufacturer’s protocol. After 6 h, media containing miR-132 mimics (50 nm), miR-132 inhibitor (100 nm), and Shh and TUG1 siRNAs (50 nm) were replaced by new medium and incubated for a further 48 h. RT-PCR was used to detect changes before and after transfection.

### Construction of expression vectors and luciferase reporter assay

The coding sequences of Shh and TUG1 were amplified and cloned into a lentiviral expression vector to generate pCDH-Shh and pCDH-TUG1. For luciferase reporter assays, the full length 3′-UTRs of Shh and TUG1 were produced by annealing the sense strand from the XhoI and Not I sites. Mutation was performed using a mutation kit (NEB, Ipswich, Canada). HEK-293 cells were cultured in six-well plates and transfected with miR-132 control or mimics (50 nM) and wild-type (WT) or mutant (Mut) Shh and TUG1 using Lipofectamine 3000. After 48 h of transfection, a Dual-Luciferase Reporter System (Promega, Madison, WI, USA) was used to evaluate the relative luciferase activity of HEK-293 cells according to the manufacturer’s protocol. Luciferase activity was normalized to Renilla luciferase activity.

### RNA extraction and quantitative real-time PCR

Total RNA was extracted using the Trizol reagent (Invitrogen) or a miRNeasy Mini Kit (Qiagen, Valencia, CA, USA) followed by reverse transcription. The ABIPRISM 7900 Sequence Detection System (Applied Biosystems, Foster City, CA, USA) was used to amplify and detect cDNA with the SYBR Green Real-time PCR Master Mix (Takara, Dalian, China). The relative expression level of miR-132, Shh, or TUG1 was normalized to U6 or GAPDH, and the results were calculated the by 2^-ΔΔCt^ method. Primers used in the experiment are shown in Table 1.

**Table 1:** Nucleotide sequences of primers used for qRT-PCR

Gene		Primer sequence (5’—3’)
*Shh*	Forward	CTCGCTGCTGGTATGCTCG
	Reverse	ATCGCTCGGAGTTTCTGGAGA
*miR-132*	Forward	GGCAACCGTGGCTTTGGA
	Reverse	TTTGGCACTAGCACATT
*TUG1*	Forward	TAACAGCCCTCCACTCCAGAT
	Reverse	AGGCACCAGCTTCAAAACCC
*U6*	Forward	AAAGCAAATCATCGGACGACC
	Reverse	GTACAACACATTGTTTCCTCGGA
*GAPDH*	Forward	GGAGCGAGATCCCTCCAAAAT
	Reverse	GGCTGTTGTCATACTTCTCATGG

### Western blot analysis

Cells and tissues were lysed in RIPA buffer, centrifuged, and the protein concentration of the supernatant was measured. Equal amounts of protein were separated by sodium dodecyl sulfate-polyacrylamide gel electrophoresis at 80 V and transferred to polyvinylidene fluoride membranes. Membranes were blocked in 5% nonfat dried milk to eliminate non-specific binding, and then incubated in primary antibodies (CST, MA, USA) at 4°C overnight. Membranes were then incubated in the corresponding fluorescence labeled anti-rabbit or anti-mouse IgG secondary antibodies and bands were visualized using the Odyssey two-color infrared laser imaging system.

### Pathological assessments and immunohistochemistry

Paraffin embedded tissue blocks were cut at a thickness of 5 μm and stained with hematoxylin and eosin (HE) for pathological assessment. Fixed sections were dewaxed and hydrated before antigens recovery by repeated cooling and heating. Sections were incubated in the indicated primary and secondary antibodies for detection of positive particles by diaminobenzidine (DAB). Color development was observed using a digital camera (Olympus, Tokyo, Japan) and calculated using Image-Pro Plus software 6.0 (Media Cybernetics, Silver Spring, MD, USA).

### Cell cycle and apoptosis analysis

Cells were treated as indicated, harvested, and washed in PBS. For cell cycle analysis, cells were suspended in anhydrous ethanol and stored at −20°C. Binding buffer containing propidium iodide (PI), NP-40, and RNaseA (BD Biosciences, San Diego, CA, USA) was added and incubated for 15 min at room temperature. For cell apoptosis analysis, cells were stained with Annexin-V and PI (BD Biosciences) for 15 min at 4°C. Cell cycle distribution and apoptosis were analyzed by flow cytometry and the results were calculated using ModFit LT software (Verity Software House, Topsham, ME, USA).

### Animal experiments

A total of 20 4-week-old male athymic BALB/c nu/nu mice (n = 5 per group) were obtained from Shanghai SLAC Laboratory Animal Co. Ltd. (Shanghai, China). All experimental animal procedures were performed strictly in accordance with the Guide for the Care and Use of Laboratory Animals, and approved by the Animal Care and Use Committee of the Shanghai Tenth People’s Hospital.

The mice were randomly divided into four groups (five in each group) as follows: Group I, vehicle; Group II, siTUG1; Group III, miR-132 mimics; Group IV; siTUG1+miR-132 inhibitor. LM3 cells were transfected with si-TUG1 or miR-132 mimics using Lipofectamine 3000. After 48 h, LM3 HCC cells were collected and subcutaneously injected into the upper flank region of all mice at a density of 5 × 10^6^ (100 μL). Mouse weights and tumor volumes were measured every 5 days. At 21 days after injection, the tumor mass and related tissues were removed and used for further analysis.

### TUNEL staining

Sections from formalin fixed, paraffin-embedded tumors were dewaxed and rehydrated for 5–10 min. The slides were treated for 15 min with Proteinase K without DNase at a concentration of 20 μg/ml according to the manufacturer’s protocols (Roche, Mannheim, Germany). The buffer was dropped into the slices and the reaction was visualized using fluorescence microscopy.

### Statistical analysis

Data were expressed as the mean ± SD, and SPSS20.0 (IBM, Chicago, IL, USA) was used to analyze the statistical significance of differences. The relationship between miR-132 and Shh or TUG1 mRNA expression was calculated by the Spearman’s test and normality (and homogeneity of variance) assumptions were used for parametric tests. Quantitative data of at least three experiments were compared using one-way analysis of variance and the differences between groups were compared using the Student’s t-test. P values of <0.05 were considered statistically significant.

## References

[1] Yang JD, Roberts LR (2010). Hepatocellular carcinoma: a global view. Nat Rev Gastroenterol Hepatol.

[2] Bosch FX, Ribes J, Diaz M, Cleries R (2004). Primary liver cancer: worldwide incidence and trends. Gastroenterology.

[3] Bangs F, Anderson KV (2016). Primary cilia and mammalian Hedgehog signaling. Cold Spring Harb Perspect Biol.

[4] Chung SI, Moon H, Ju HL, Cho KJ, Kim do Y, Han KH, Eun JW, Nam SW, Ribback S, Dombrowski F, Calvisi DF, Ro SW (2016). Hepatic expression of Sonic Hedgehog induces liver fibrosis and promotes hepatocarcinogenesis in a transgenic mouse model. J Hepatol.

[5] Bouscary D (2016). Rational for targeting the hedgehog signalling pathway in acute myeloid leukemia with FLT3 mutation. Ann Transl Med.

[6] Habib JG, O’Shaughnessy JA (2016). The hedgehog pathway in triple-negative breast cancer. Cancer Med.

[7] Ruch JM, Kim EJ (2013). Hedgehog signaling pathway and cancer therapeutics: progress to date. Drugs.

[8] Abidi A (2014). Hedgehog signaling pathway: a novel target for cancer therapy: vismodegib, a promising therapeutic option in treatment of basal cell carcinomas. Indian J Pharmacol.

[9] Cao T, Li H, Hu Y, Ma D, Cai X (2014). miR-144 suppresses the proliferation and metastasis of hepatocellular carcinoma by targeting E2F3. Tumour Biol.

[10] Cai H, Xue Y, Wang P, Wang Z, Li Z, Hu Y, Li Z, Shang X, Liu Y (2015). The long noncoding RNA TUG1 regulates blood-tumor barrier permeability by targeting miR-144. Oncotarget.

[11] Dai W, Wang C, Wang F, Wang Y, Shen M, Chen K, Cheng P, Zhang Y, Yang J, Zhu R, Zhang H, Li J, Zheng Y (2014). Anti-miR-197 inhibits migration in HCC cells by targeting KAI 1/CD82. Biochem Biophys Res Commun.

[12] Zhao N, Koenig SN, Trask AJ, Lin CH, Hans CP, Garg V, Lilly B (2015). MicroRNA miR145 regulates TGFBR2 expression and matrix synthesis in vascular smooth muscle cells. Circ Res.

[13] Li R, Chung AC, Dong Y, Yang W, Zhong X, Lan HY (2013). The microRNA miR-433 promotes renal fibrosis by amplifying the TGF-beta/Smad3-Azin1 pathway. Kidney Int.

[14] Yu F, Zheng Y, Hong W, Chen B, Dong P, Zheng J (2015). MicroRNA200a suppresses epithelialtomesenchymal transition in rat hepatic stellate cells via GLI family zinc finger 2. Mol Med Rep.

[15] Yu F, Lu Z, Huang K, Wang X, Xu Z, Chen B, Dong P, Zheng J (2016). MicroRNA-17-5p-activated Wnt/beta-catenin pathway contributes to the progression of liver fibrosis. Oncotarget.

[16] Kumar MM, Goyal R (2017). LncRNA as a therapeutic target for angiogenesis. Curr Top Med Chem.

[17] Shi L, Peng F, Tao Y, Fan X, Li N (2016). Roles of long noncoding RNAs in hepatocellular carcinoma. Virus Res.

[18] Yu TT, Xu XM, Hu Y, Deng JJ, Ge W, Han NN, Zhang MX (2015). Long noncoding RNAs in hepatitis B virus-related hepatocellular carcinoma. World J Gastroenterol.

[19] Karreth FA, Reschke M, Ruocco A, Ng C, Chapuy B, Leopold V, Sjoberg M, Keane TM, Verma A, Ala U, Tay Y, Wu D, Seitzer N (2015). The BRAF pseudogene functions as a competitive endogenous RNA and induces lymphoma *in vivo*. Cell.

[20] Xu L, Dai W, Li J, He L, Wang F, Xia Y, Chen K, Li S, Liu T, Lu J, Zhou Y, Wang Y, Guo C (2016). Methylation-regulated miR-124-1 suppresses tumorigenesis in hepatocellular carcinoma by targeting CASC3. Oncotarget.

[21] Lv J, Fan HX, Zhao XP, Lv P, Fan JY, Zhang Y, Liu M, Tang H (2016). Long non-coding RNA Unigene56159 promotes epithelial-mesenchymal transition by acting as a ceRNA of miR-140-5p in hepatocellular carcinoma cells. Cancer Lett.

[22] Sicklick JK, Li YX, Jayaraman A, Kannangai R, Qi Y, Vivekanandan P, Ludlow JW, Owzar K, Chen W, Torbenson MS, Diehl AM (2006). Dysregulation of the Hedgehog pathway in human hepatocarcinogenesis. Carcinogenesis.

[23] Jung Y, McCall SJ, Li YX, Diehl AM (2007). Bile ductules and stromal cells express hedgehog ligands and/or hedgehog target genes in primary biliary cirrhosis. Hepatology.

[24] Huang S, He J, Zhang X, Bian Y, Yang L, Xie G, Zhang K, Tang W, Stelter AA, Wang Q, Zhang H, Xie J (2006). Activation of the hedgehog pathway in human hepatocellular carcinomas. Carcinogenesis.

[25] Chen YJ, Lin CP, Hsu ML, Shieh HR, Chao NK, Chao KS (2011). Sonic hedgehog signaling protects human hepatocellular carcinoma cells against ionizing radiation in an autocrine manner. Int J Radiat Oncol Biol Phys.

[26] Liu K, Li X, Cao Y, Ge Y, Wang J, Shi B (2015). MiR-132 inhibits cell proliferation, invasion and migration of hepatocellular carcinoma by targeting PIK3R3. Int J Oncol.

[27] Zhang X, Tang W, Li R, He R, Gan T, Luo Y, Chen G, Rong M (2016). Downregulation of microRNA-132 indicates progression in hepatocellular carcinoma. Exp Ther Med.

[28] Mokutani Y, Uemura M, Munakata K, Okuzaki D, Haraguchi N, Takahashi H, Nishimura J, Hata T, Murata K, Takemasa I, Mizushima T, Doki Y, Mori M (2016). Down-regulation of microRNA-132 is associated with poor prognosis of colorectal cancer. Ann Surg Oncol.

[29] Qin J, Ke J, Xu J, Wang F, Zhou Y, Jiang Y, Wang Z (2015). Downregulation of microRNA-132 by DNA hypermethylation is associated with cell invasion in colorectal cancer. Onco Targets Ther.

[30] Liu X, Yan S, Pei C, Cui Y (2015). Decreased microRNA-132 and its function in human non-small cell lung cancer. Mol Med Rep.

[31] Fukao A, Aoyama T, Fujiwara T (2015). The molecular mechanism of translational control via the communication between the microRNA pathway and RNA-binding proteins. RNA Biol.

[32] Salmena L, Poliseno L, Tay Y, Kats L, Pandolfi PP (2011). A ceRNA hypothesis: the Rosetta Stone of a hidden RNA language?. Cell.

[33] Wang W, Zhuang Q, Ji K, Wen B, Lin P, Zhao Y, Li W, Yan C (2017). Identification of miRNA, lncRNA and mRNA-associated ceRNA networks and potential biomarker for MELAS with mitochondrial DNA A3243G mutation. Sci Rep.

[34] Yang S, Ning Q, Zhang G, Sun H, Wang Z, Li Y (2016). Construction of differential mRNA-lncRNA crosstalk networks based on ceRNA hypothesis uncover key roles of lncRNAs implicated in esophageal squamous cell carcinoma. Oncotarget.

[35] Sui J, Li YH, Zhang YQ, Li CY, Shen X, Yao WZ, Peng H, Hong WW, Yin LH, Pu YP, Liang GY (2016). Integrated analysis of long non-coding RNAassociated ceRNA network reveals potential lncRNA biomarkers in human lung adenocarcinoma. Int J Oncol.

[36] Lu MH, Tang B, Zeng S, Hu CJ, Xie R, Wu YY, Wang SM, He FT, Yang SM (2015). Long noncoding RNA BC032469, a novel competing endogenous RNA, upregulates hTERT expression by sponging miR-1207-5p and promotes proliferation in gastric cancer. Oncogene.

[37] Yu F, Lu Z, Cai J, Huang K, Chen B, Li G, Dong P, Zheng J (2015). MALAT1 functions as a competing endogenous RNA to mediate Rac1 expression by sequestering miR-101b in liver fibrosis. Cell Cycle.

[38] Collins JF (2015). Long noncoding RNAs and hepatocellular carcinoma. Gastroenterology.

